# Spectral-conversion film potential for greenhouses: Utility of green-to-red photons conversion and far-red filtration for plant growth

**DOI:** 10.1371/journal.pone.0281996

**Published:** 2023-02-23

**Authors:** Yujin Park, Erik S. Runkle

**Affiliations:** 1 Department of Horticulture, Michigan State University, East Lansing, Michigan, United States of America; 2 College of Integrative Sciences and Arts, Arizona State University, Tempe, Arizona, United States of America; Mohanlal Sukhadia University, INDIA

## Abstract

Although green (G, 500 to 600 nm) and far-red (FR, 700 to 800 nm) light play important roles in regulating plant growth and development, they are often considered less useful at stimulating photosynthesis than red (R, 600 to 700 nm) and blue (B, 400 to 500 nm) light. Based on this perception, approaches to modifying the transmission of greenhouse glazing materials include (1) conversion of G photons from sunlight into R photons and (2) exclusion of the near-infrared (>700 nm) fraction of sunlight. We evaluated these approaches using simulated scenarios with light-emitting diodes to determine how partial and complete substitution of G with R light and exclusion of FR light affected the growth of lettuce and tomato grown indoors. The substitution of G with R light had little or no effect on fresh and dry mass of tomato. However, with the presence of FR light, fresh and dry mass of lettuce increased by 22–26% as G light was increasingly substituted with R light. In tomato, excluding FR inhibited plant height, leaf area, and dry mass by 60–71%, 10–37%, and 20–44%, respectively. Similarly, in lettuce, excluding FR inhibited plant diameter, leaf length, and dry mass by 15–23%, 23–33%, or 28–48%, respectively. We conclude that the spectral conversion of G-to-R photons can promote plant growth in at least some crop species, such as lettuce, while the exclusion of FR decreases crop growth and yield.

## 1. Introduction

The photon spectrum (or light quality) refers to the number or portion of photons at each wavelength or waveband, including blue (B, 400–500 nm), green (G, 500–600 nm), red (R, 600–700 nm), and far-red (FR, 700–800 nm) light [1]. For sunlight, the proportion of B, G, R, and FR photons between 400 nm and 800 nm is 23%, 26%, 26%, and 25%, respectively [2]. Photoselective or spectral conversion films as covering materials or shade curtains make it possible to manipulate the solar spectrum inside greenhouses [2–15]. In addition, recent advances with wavelength-selective and (semi-) transparent photovoltaic systems allow transmission of specific portions of the solar spectrum for plant growth, while the systems can capture the remaining radiation to generate electricity [16–20].

An objective of solar spectral manipulation for greenhouse applications has been to transmit the most effective regions of the light spectrum for photosynthesis and plant growth. The selection criteria of effective light spectra for photosynthesis and plant growth have been based on the definition of photosynthetically active radiation (PAR) [7, 8, 13, 16], the quantum yield for photosynthesis [6, 14, 18, 20], or the absorption spectrum of chlorophylls [3, 9, 10, 12, 20]. The current definition of PAR refers to the waveband from 400 to 700 nm (excluding FR light), which is considered the effective spectral range used for plant photosynthesis [21]. Isolated and purified chlorophyll *a* and *b* absorb B and R light strongly (with the peak absorption at 428 nm and 661 nm for chlorophylls *a* and 470 nm and 642 nm for chlorophylls *b*) and G and FR light weakly [22]. Based on absorbed light, the relative instantaneous quantum yield of R photons (0.91) for CO_2_ fixation is higher than that of G (0.87), B (0.73), or FR (0.12) photons [23, 24]. Thus, two conventional paradigms are 1) FR photons are slightly or not effective at stimulating photosynthesis and 2) G photons are less efficient than B or especially R photons for photosynthesis and thus, plant growth.

These two paradigms have led to two general approaches to manipulate transmission of the solar spectrum inside greenhouses. First, some greenhouse covering materials partially block near-infrared (NIR, 700–1000 nm) to decrease the temperature rise inside the greenhouse, especially during the summer in warm climates [7, 16, 17, 25–27]. Second, several spectral conversion films (used for greenhouse covering materials and luminescent solar concentrators) convert G light into R light by using luminescent materials, including organic dyes and inorganic phosphors, which absorb G light and fluoresce it as R light [3, 9, 10, 12, 28].

There are several potential faults to these two simplistic paradigms. First, the action spectrum of photosynthesis and absorption spectra of chlorophylls were developed based on instantaneous measurements of a single leaf or extracted and purified chloroplasts. They consider the effects of independent wavelengths and do not consider the synergetic interaction between FR and shorter wavelengths, nor the effects of the photon spectrum on whole-canopy photosynthesis, plant morphological or acclimation changes over time, or light-absorbing accessory pigments [29, 30]. In addition, accumulating evidence shows FR photons (specifically 700–750 nm) are equally photosynthetically active when they are applied with photons within PAR, and thus, it has proposed to change the definition of PAR to include FR photons to better predict photosynthesis [31–34]. In sunflower (*Helianthus annuu*s ‘Mammoth’), corn (*Zea mays* ‘Early Sunglow’), burdock (*Arctium minus*), and Norway maple (*Acer platanoides*), accounting for the absorbed 400–750 nm photons estimated the quantum yield of photosynthesis more accurately under sunlight (with and without FR photon filtration) than PAR, as the quantum yield of photosynthesis was overestimated based on the absorbed 400–700 nm photons [34].

A second limitation of these paradigms is that G photons contribute to leaf and canopy-level photosynthesis. At leaf level, as the scattering of G light (i.e., the detour effect) increases, an increasing fraction can be absorbed by plant leaves [35–37]. The absorbed G photons drive photosynthesis with a comparable quantum yield as R photons [23]. At the canopy level, at least some G photons transmitted through or reflected by leaves at the upper part of the plant canopy can be used for photosynthesis by leaves at the lower part of the canopy [38–40].

Third, G and FR light can induce shade-avoidance responses, including promotion of stem elongation and leaf expansion, which increase light capture of a plant canopy [39–42]. The G-light induced shade-avoidance response is mediated at least partly by inactivating cryptochromes [43] and by an unknown G-light-signaling pathway independent of phytochromes and cryptochromes [41, 44]. Phytochromes are the primary photoreceptors that mediate FR-light induced shade-avoidance responses. FR light inactivates phytochromes while R light activates them. Thus, increasing FR relative to R light or the FR fraction (FR/R+FR) [45] decreases the fraction of active phytochromes, or the phytochrome photoequilibrium (PPE), which typically increases the magnitude of shade-avoidance responses. For example, in geranium (*Pelargonium hortorum*) and snapdragon (*Antirrhinum majus*) seedlings, as PPE decreased with increasing FR fraction, stem elongation and leaf expansion increased linearly [46]. Several studies showed that increases in leaf area and subsequent light capture contributed to increased plant biomass gain more than increases in instantaneous photosynthesis, including in arabidopsis (*Arabidopsis thaliana*) and cucumber (*Cucumis sativus*) [47, 48]. Therefore, when the effects of G and FR light on plant morphology and photosynthesis are not considered, G and FR light contributions to plant growth can be underestimated. Collectively, these challenge the basis of photon-selective and photon-conversion films and whether they can increase (or at least not decrease) crop productivity.

In this study, we simulated “best case” scenarios of spectral conversion or exclusion of sunlight using light-emitting diodes (LEDs) to determine how partial and full substitution of G with R light, with or without exclusion of FR light, influence plant morphology and shoot biomass. We postulated that substituting G with R light and excluding FR light would suppress shoot biomass accumulation mainly by inhibiting stem elongation and leaf expansion.

## 2. Materials and methods

### 2.1. Plant material and propagation conditions

We performed the experiment twice in a refrigerated walk-in growth room of the Controlled-Environment Lighting Laboratory (Michigan State University, East Lansing, MI) described by Zhang et al. [49]. Seeds of red-leaf lettuce (*Lactuca sativa*) ‘Cherokee’ and indeterminate tomato (*Solanum lycopersicum*) ‘Bamborange’ were obtained from seed producers (Johnny’s Selected Seeds, Winslow, ME; and Syngenta, Gilroy, CA, respectively). In each of two replications, we germinated the seeds of lettuce and tomato in a rockwool substrate following the method previously described by Meng et al. [50]. The seeded rockwool substrate in a plastic tray was placed on a growing rack under an 18-h photoperiod with a photosynthetic photon flux density (PPFD; 400–700 nm) of 180 μmol∙m^–2^∙s^–1^ from warm-white (peak = 639 nm and correlated color temperature = 2700 K) LEDs. The PPFD delivered from warm-white LEDs was measured using a handheld light meter (LI-250A; LI-COR, Inc., Lincoln, NE) based on an average from nine measurements made at predetermined horizontal positions at the substrate surface (54 cm below the LED fixtures). The seeded substrate was covered with a transparent humidity dome and kept wet for the initial 4 d (for lettuce) or 5 d (for tomato) until complete germination. After germination, we irrigated seedlings as needed (every one or two days) through subsurface irrigation with deionized water supplemented with a water-soluble fertilizer (12N–4P_2_O_5_–16K_2_O RO Hydro FeED; JR Peters, Inc., Allentown, PA) and magnesium sulfate (Epsom salt; Pennington Seed, Inc., Madison, GA) to provide the following nutrients (in mg∙L^-1^): 125 N, 18 P, 138 K, 73 Ca, 49 Mg, 39 S, 1.7 Fe, 0.52 Mn, 0.56 Zn, 0.13 B, 0.47 Cu, and 0.13 Mo. We measured the EC and pH of the nutrient solution each day with a pH and electrical conductivity meter (HI9814; Hanna Instruments, Woonsocket, RI) and maintained EC and pH at 1.0–1.2 mS∙cm^–1^ and 5.5–5.8, respectively. We adjusted the pH using potassium bicarbonate or diluted (1:31) 95–98% sulfuric acid (J.Y. Baker, Inc., Philipsburg, NJ).

Seeds and seedlings were germinated and grown at an air temperature setpoint of 22°C and ambient CO_2_. Air temperature and PPFD near plant height and the ambient CO_2_ concentration and relative humidity of the growth room were monitored and recorded as described by Meng et al. [50]. During two replications, the mean air temperature, CO_2_ concentration, and relative humidity (± standard deviation) in the growth room during propagation were 22.2 ± 0.4°C, 386 ± 29 ppm, and 53% ± 11%, respectively.

### 2.2. Simulated lighting treatments and growth conditions

In both replications, ten days after seed sow (when plants had fully expanded cotyledons and as the first and second true leaves were emerging), 15 seedlings of each species in rockwool cubes were transplanted into a deep-flow technique hydroponic system as described by Meng et al. [51] under each of nine lighting treatments. Plant roots were fully submerged in a nutrient solution made with deionized water supplemented with the same water-soluble fertilizer and magnesium sulfate as for seedlings to supply the following nutrients (in mg∙L^-1^): 150 N, 22 P, 166 K, 88 Ca, 58 Mg, 47 S, 2.1 Fe, 0.63 Mn, 0.68 Zn, 0.15 B, 0.56 Cu, and 0.15 Mo. The EC, pH, and water temperature of the nutrient solution for each rack were measured daily in each reservoir with a pH and electrical conductivity meter (HI9814; Hanna Instruments). When pH was outside 5.5–5.8, it was adjusted using potassium bicarbonate or diluted (1:31) 95–98% sulfuric acid (J.Y. Baker, Inc.). During two replications, the mean EC, pH, and water temperature of the nutrient solutions for all lighting treatments were 1.7 ± 0.1 mS∙cm^–1^, 5.8 ± 0.7, and 23.6 ± 0.7°C, respectively. Plants were grown at an air temperature setpoint of 22°C and ambient relative humidity and CO_2_. The environmental data were monitored and collected as described previously for seedlings. During two replications, the mean air temperature, CO_2_ concentration, and relative humidity in the growth room were 21.7 ± 0.7°C, 388 ± 32 ppm, and 51% ± 6%, respectively.

**Fig 1:**
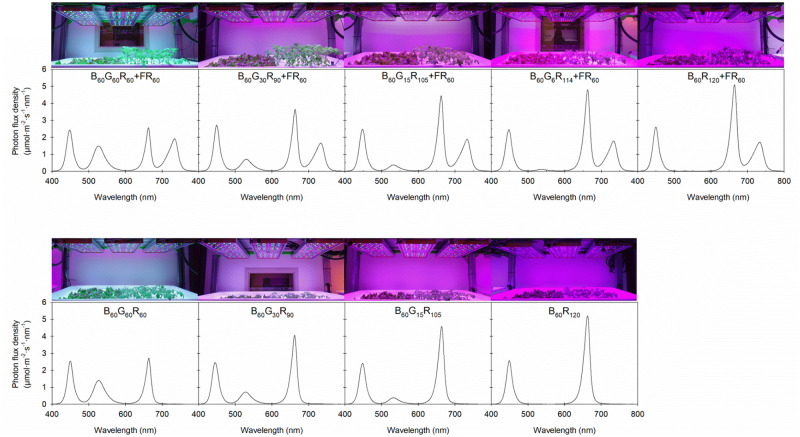
The spectra of nine lighting treatments. Lighting treatments consisted of blue (B, 400–500 nm), green (G, 500–600 nm), red (R, 600–700 nm), and far-red (FR, 700–800 nm) light delivered by light-emitting diodes. The value that follows each waveband indicates its photon flux density in μmol∙m^−2^∙s^−1^.

Each of the nine lighting treatments was provided by LED fixtures as previously described that delivered a PPFD of 180 μmol∙m^−2^∙s^−1^ and an 18-h photoperiod (0500–2300 HR). The treatments delivered the following photon flux densities (subscript in μmol∙m^−2^∙s^−1^) of B, G, R, or/and FR light: B_60_G_60_R_60_FR_60_, B_60_G_30_R_90_FR_60_, B_60_G_15_R_105_FR_60_, B_60_G_6_R_114_FR_60_, B_60_R_120_FR_60_, B_60_G_60_R_60_, B_60_G_30_R_90_, B_60_G_15_R_105_, and B_60_R_120_. The nine lighting treatments were randomly allocated to a tier among three growing racks in a growth room at each replication. Lighting treatments were randomly assigned and carried out separately in each replication to mitigate any positional effects inside the growth room. For each lighting treatment, the photon flux densities of B (peak = 449 nm), G (peak = 526 nm), R (peak = 664 nm), and FR (peak = 733 nm) LEDs were independently adjusted following the method described by Meng et al. [51] (Fig 1). The photon flux density of each waveband was calculated using 100-nm wavebands; the yield photon flux density (YPFD) was calculated as described by Sager et al. [24]; and internal PPE (iPPE) was calculated following Kusuma and Bugbee [52], which incorporates spectral distortion within a leaf (Table 1).

**Table 1:** Photon flux density and spectral properties of nine lighting treatments. Lighting treatments consisted of blue (B, 400–500 nm), green (G, 500–600 nm), red (R, 600–700 nm), and far-red (FR, 700–800 nm) light delivered by light-emitting diodes. The value that follows each waveband indicates its photon flux density in μmol∙m^−2^∙s^−1^.

Lighting treatment	PPFD^1^ (400–700 nm)	% G^2^	TPFD^3^ (400–800 nm)	YPFD^4^	iPPE^5^	FR/(R+FR)^6^
B_60_G_60_R_60_+FR_60_	184.5	33.3	244.0	160.4	0.31	0.51
B_60_G_30_R_90_+FR_60_	178.9	16.6	232.2	158.8	0.39	0.40
B_60_G_15_R_105_+FR_60_	179.2	8.3	237.6	162.7	0.41	0.38
B_60_G_6_R_114_+FR_60_	178.6	3.3	234.0	163.0	0.44	0.34
B_60_R_120_+FR_60_	186.1	0	241.9	169.8	0.46	0.33
B_60_G_60_R_60_	178.8	33.3	179.7	146.4	0.81	0.02
B_60_G_30_R_90_	181.4	16.6	182.7	153.2	0.83	0.02
B_60_G_15_R_105_	180.9	8.3	182.2	155.0	0.84	0.01
B_60_R_120_	179.6	0	180.7	155.8	0.85	0.01

^1^Photon flux densities (in μmol∙m^−2^∙s^−1^) were integrated from 400 nm to 700 nm as photosynthetic photon flux density (PPFD).

^2^Calculated as percentage of G photon flux density relative to PPFD.

^3^Total photon flux densities (TPFD, in μmol∙m^−2^∙s^−1^) were integrated from 400 nm to 800 nm.

^4^The product of TPFD and relative quantum efficiency was calculated as yield photon flux density (YPFD) based on McCree [23] and Sager et al. [24].

^5^Estimated internal phytochrome photoequilibria (iPPE) was calculated following Kusuma and Bugbee [52].

^6^FR fraction, or ratio of photon flux integral of FR (700–800 nm) to the sum of the photon flux integral of R (600–700 nm) and FR, was calculated following Kusuma and Bugbee [45].

### 2.3. Data collection

Ten random plants per species were selected from each treatment and replication and used for data collection on the following number of days after seed sow (rep 1, 2): lettuce (30, 31) and tomato (28, 27). The following data were collected in each plant of each species: leaf number, shoot fresh and dry mass [using an analytical balance (GX-1000; A&D Store, Inc., Wood Dale, IL)], and soil and plant analyzer development (SPAD) value, as an index of relative leaf chlorophyll concentration [using a portable chlorophyll meter (SPAD-502; Konica Minolta Sensing, Inc., Chiyoda, Tokyo, Japan)]. In addition, plant diameter, length and width of the fifth-most mature leaf, and leaf color [using a colorimeter (Chroma Meter CR-400; Konica Minolta Sensing, Inc.)] were measured in lettuce, and leaf area [using a leaf-area meter (LI-3000; LI-COR, Lincoln, NE)] was measured in tomato. (Leaf area of lettuce was not measured because their ruffled character prevents accurate measurement.) Leaves with a length of ≥ 5 cm for lettuce or 2 cm for tomato (from the base of the petiole to the tip of the terminal leaflet) were counted in leaf number and included in leaf area measurements. Shoot dry mass was measured after the plants were abscised at the growing substrate surface and dried in an oven at ≥66°C for ≥5 d (model 630; Napco Scientific Company, Tualatin, OR). Shoot moisture content (%) was calculated as the difference between shoot fresh and dry mass per shoot fresh mass following Meng et al. [50]. As a non-destructive measurement of leaf relative chlorophyll concentration, the SPAD index was measured thrice for each plant, between the midrib and the leaf margin of the fifth most-mature leaf in lettuce or the third or fourth most-mature leaf in tomato.

### 2.4. Statistical analysis

This experiment was conducted as a randomized complete block design with replication (block), lighting treatment (experimental unit), and plant per species (subsample). Data from two replications were pooled and analyzed with SAS (version 9.4; SAS Institute, Inc., Cary, NC). We determined the main effects of the substitution of G with R light (or percentage of G light) and presence of FR light and the interaction effect of percentage of G light and presence of FR light by analysis of variance (ANOVA) on lettuce or tomato using the SAS PROC MIXED procedure [with two fixed factors (percentage of G light and photon flux density of FR light) and two random factors (blocks and interaction between blocks, percentage of G light, and photon flux density of FR light)] (Table 2). We modeled the effects of the percentage of G light on plant growth, with or without FR light, using linear regression by SAS PROC REG. Simple linear regression analysis for each species included 100 data points (2 replications × 5 G light percentages × 10 plants/treatment/replication) with FR light or 80 data points (2 replications × 4 G light percentages × 10 plants/treatment/replication) without FR light.

**Table 2:** Results from two-factor analysis of variance. *P* values are listed for the effects of green (G, 500–600 nm) light, far-red (FR, 700–800 nm) light, or their interaction on plant growth parameters of lettuce and tomato.

	Lettuce			Tomato		
Factor	G	FR	G×FR	G	FR	G×FR
Plant diameter	0.104	<0.001	0.168	–	–	–
Plant height	–	–	–	0.070	<0.001	0.236
Leaf number	0.428	0.036	0.507	0.158	0.503	0.073
Leaf length	0.739	<0.001	0.277	–	–	–
Leaf width	0.598	<0.001	0.319	–	–	–
Leaf area	–	–	–	0.589	0.001	0.272
Shoot fresh mass	0.019	<0.001	0.017	0.384	<0.001	0.359
Shoot dry mass	0.050	<0.001	0.008	0.369	<0.001	0.236
Shoot moisture content	0.754	0.563	0.432	0.868	<0.001	0.348
SPAD index	0.080	0.095	0.194	0.002	<0.001	0.225

## 3. Results

### 3.1. Plant morphology

**Fig 2:**
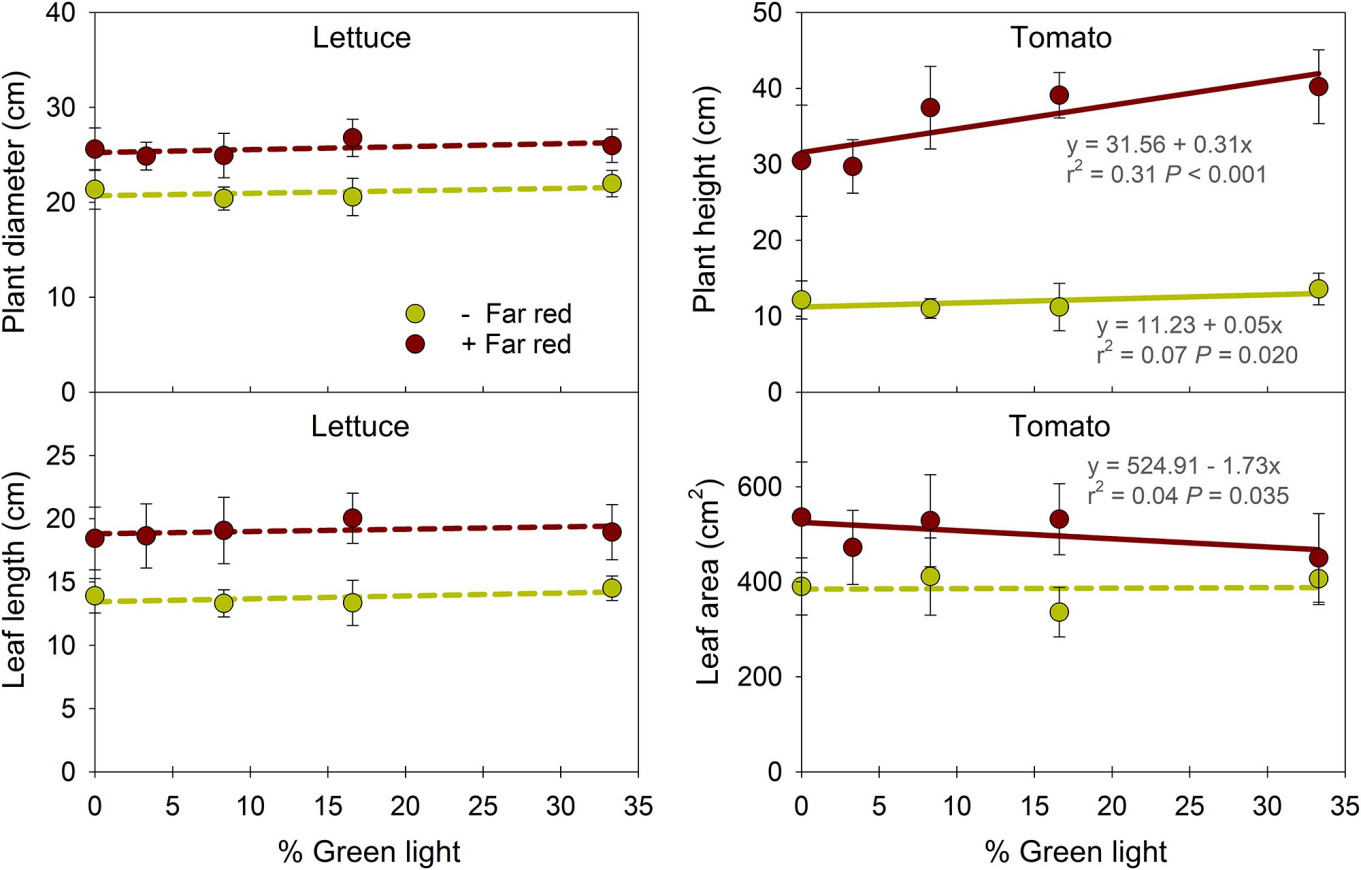
Influence of percentage of green light on plant diameter and leaf length of the fifth-most mature leaf of lettuce ‘Cherokee’ and plant height and leaf area of tomato ‘Bamborange’ with (+) and without (-) far-red light. See Fig 1 and Table 1 for information on the lighting treatments. Each data point represents the mean and standard deviation of two replications with 10 subsamples (plants) per replication and species (n = 20). Regression equations, r^2^ values, and *P* values are presented when statistically significant (solid line) but not when non-significant (dashed line).

Partial substitution of G with R light did not influence plant diameter or leaf length of lettuce (Fig 2). However, plant height of tomato increased as the percentage of G light in PAR increased from 0 to 33% (and as the R percentage decreased), regardless of FR light. The increase in plant height was greater with FR light (32%) than without (12%). With FR light, leaf area in tomato decreased by 16% as G light increased from 0 to 33%. At the same portion of G light, excluding FR light decreased plant diameter (15–23%) and leaf length (23–33%) of lettuce and plant height (60–71%) and leaf area (10–37%) of tomato.

### 3.2. Leaf number and relative chlorophyll concentration

**Fig 3:**
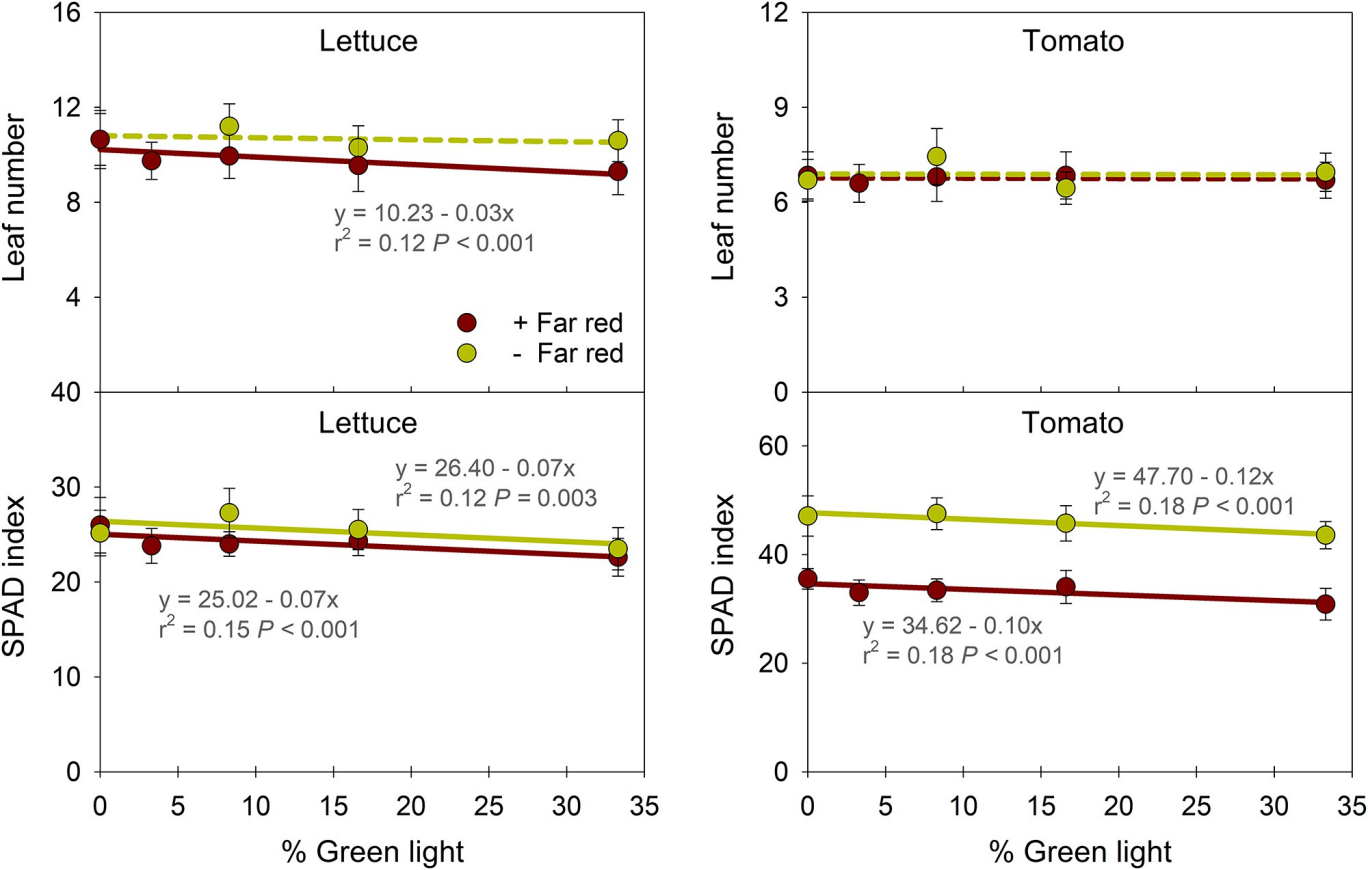
Influence of percentage of green light on leaf number and SPAD index of lettuce ‘Cherokee’ and tomato ‘Bamborange’ with (+) and without (-) far-red light. See Fig 1 and Table 1 for information on the lighting treatments. Each data point represents the mean and standard deviation of two replications with 10 subsamples (plants) per replication and species (n = 20). Regression equations, r^2^ values, and *P* values are presented when statistically significant (solid line) but not when non-significant (dashed line).

Lettuce leaf number decreased by 0.9–1.3 as the percentage of G light in PAR increased from 0 to 33% with FR light, but not without (Fig 3). On average, lettuce had 0.8 more leaves without FR light than with it. SPAD index of lettuce and tomato grown with and without FR light decreased (by 13% and 7–8%, respectively) as G light increased from 0 to 33%. At the same portion of G light, SPAD index of tomato was 33–42% greater without FR light than with it. Lighting treatments had little to no effect on leaf number of tomato.

### 3.3. Shoot fresh and dry mass

**Fig 4:**
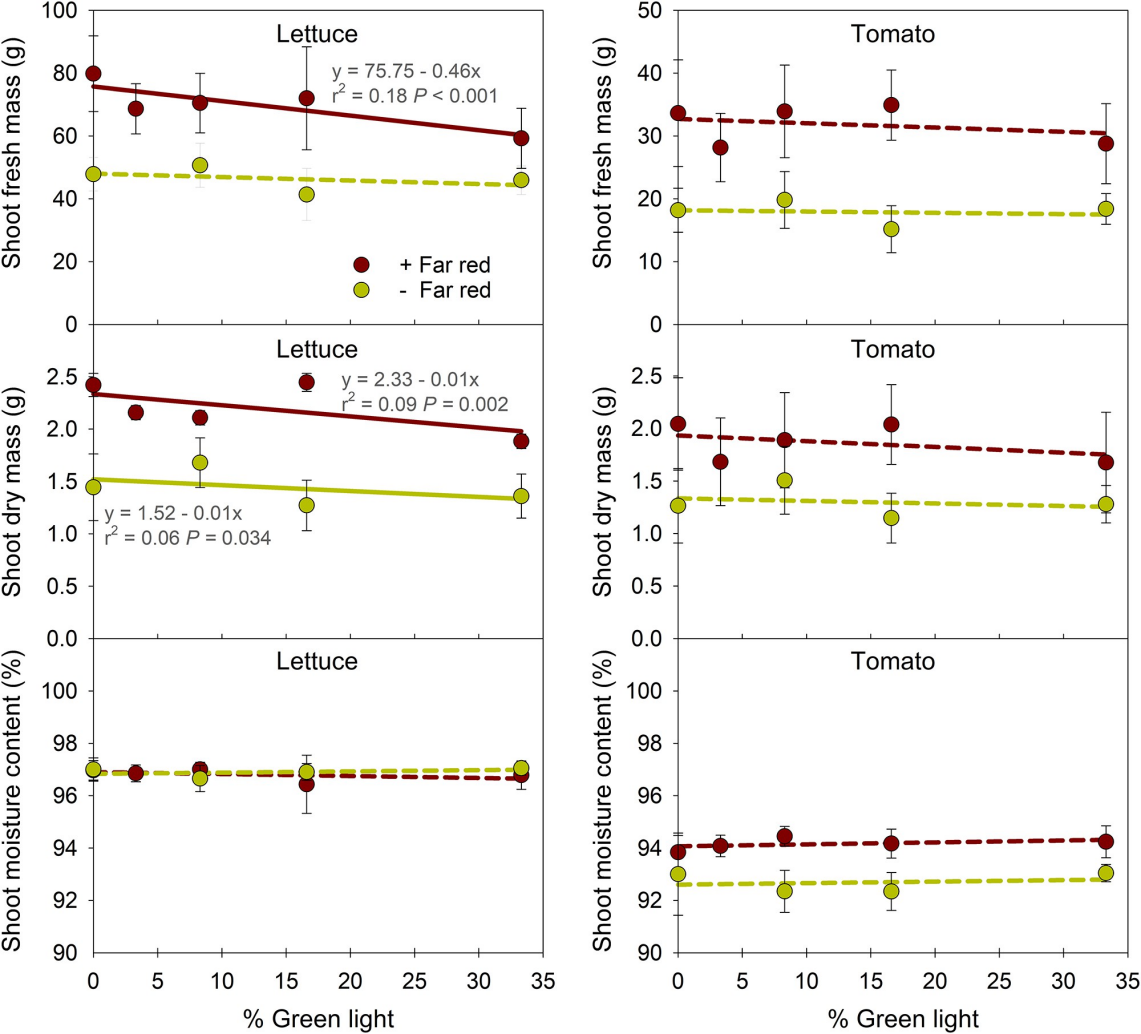
Influence of percentage of green light on shoot fresh mass, dry mass, and moisture content of lettuce ‘Cherokee’ and tomato ‘Bamborange’ with (+) and without (-) far-red light. See Fig 1 and Table 1 for information on the lighting treatments. Each data point represents the mean and standard deviation of two replications with 10 subsamples (plants) per replication and species (n = 20). Regression equations, r^2^ values, and *P* values are presented when statistically significant (solid line) but not when non-significant (dashed line).

In lettuce, increasing the proportion of G light in PAR from 0 to 33% decreased shoot fresh mass (by 26%) and dry mass (by 22%) with FR light, but there was little effect in the absence of FR (Fig 4). In tomato, the percentage of G light in PAR did not affect shoot fresh and dry mass. At the same portion of G light, excluding FR light decreased shoot fresh and dry mass in both lettuce (by 22–43% and 28–48%, respectively) and tomato (by 36–57% and 20–44%, respectively). When shoot moisture content was calculated to compare the plant water status at harvest, lighting treatments did not influence water content in lettuce. In tomato, shoot moisture content was not influenced by the percentage of G light but, at the same portion of G light, excluding FR light decreased moisture content by 1–2%, indicating the decreases in tomato shoot fresh mass without FR light was attributed to decreases in shoot dry mass and shoot moisture content.

## 4. Discussion

### 4.1. Substituting G with R light

This study used light-emitting diodes (LEDs) to simulate a “best case” scenario with a spectral conversion film, in which G photons were replaced by R photons on a 1:1 basis. G light can induce shade-avoidance responses, including elongation of hypocotyls [43, 53], stems [54], and petioles, as well as upward leaf reorientation [41, 44]. In this study, as G was substituted with R light, stem length of tomato decreased by 24% in the presence of FR light and 11% without FR light (Fig 2). When FR was in the photon spectrum, the increase in R light (and decrease in G light) decreased the FR fraction (or increased the iPPE) from 0.51 (0.31) to 0.33 (0.46), while without FR, the substitution of G with R had little effect on the FR fraction or iPPE (Table 1). Therefore, this decrease in the FR fraction (or increase in iPPE) likely suppressed stem elongation when FR was present. Considering that increases in the FR fraction (or decreases in PPE) can decrease the leaf development rate or leaf number [55, 56], and substituting G with R light increased leaf number in lettuce only when FR was present (Fig 3), we attribute the increase in leaf number to the decrease in the FR fraction (or increases in iPPE).

Substituting G with R light slightly promoted leaf expansion in tomato in the presence of FR light but had little to no effect on leaf size in lettuce (Fig 2). In past studies, there were variable effects of the substitution of G with R light on leaf expansion. For example, substituting 30% of G with R light inhibited stem elongation in pepper (*Capsicum annum*), soybean (*Glycine max*), and lettuce at a PPFD of 200 μmol∙m^–2^∙s^–1^ and in tomato at a PPFD of 500 μmol∙m^–2^∙s^–1^ [54]. In contrast, the substitution of G with R light did not affect the leaf area index of any species except for lettuce, in which it increased leaf area. In impatiens (*Impatiens walleriana*), salvia (*Salvia splendens*), and petunia (*Petunia* × *hybrida*) seedlings, substituting 80 μmol∙m^–2^∙s^–1^ of G with R light (while B was constant at 80 μmol∙m^–2^∙s^–1^) did not influence leaf area, but it did decrease it in tomato seedlings [57]. In addition, the substitution of G with R light (e.g., 12%B+44%G+44%R vs. 12%B+88%R) at a PPFD of 224 μmol∙m^–2^∙s^–1^ did not influence leaf area of green-leaf basil (*Ocimum basilicum*), green mustard (*Brassica carinata*), green kale (*Brassica napus* var. *pabularia*), and red kale (*Brassica oleracea*) [58]. In contrast, the substitution of G with R light increased leaf area of purple-leaf basil and red mustard ‘Red Giant’ (*Brassica juncea*) [58]. In three arabidopsis wild-type accessions, leaf area and dry mass were less under amber light (31% G+49%R) than R light alone, but Col-0 and C24 showed weaker responses than Est-1, indicating genotype-specific responses to G light [59]. However, the effects of G light in some of these studies could be at least partly attributed to simultaneous changes to B light. In lettuce ‘Rouxai’, substituting 60 μmol∙m^–2^∙s^–1^ of G with R light did not influence leaf length and width when B light was at 0, 20, or 60 μmol∙m^–2^∙s^–1^, but leaf width was 9% smaller when B light was at 100 μmol∙m^–2^∙s^–1^ [50]. These results suggest that the substitution of G with R light has relatively small and inconsistent effects on leaf expansion and depends on species, genotype, and additional wavelengths, particularly B light.

In this study, when FR light was present, substitution of G with R light had contrasting responses in tomato with respect to extension growth: it slightly increased leaf area yet decreased stem length. Leaf (node) number of tomato was similar among all treatments (Fig 3) and thus, internode length apparently increased with G light. Shade-induced stem elongation requires more carbon allocation to stems at the expense of leaves [55, 60]. The stem elongation of tomato with increasing G light in the presence of FR might limit carbon partitioning toward leaves, causing a decrease in leaf area. Without FR light, increasing G light slightly increased plant height but could have had little effect on carbon partitioning to the leaf and subsequently leaf growth.

The average relative quantum efficiency value for R light (0.91) is slightly greater than that of G light (0.87) [23, 24]. A few previous studies showed the promotive effects of substitution of G with R on photosynthesis and biomass accumulation. For example, leaf photosynthesis rates in lettuce ‘Buttercrunch’ and strawberry (*Fragaria* × *ananassa*) ‘Seolhyang’ increased under G-to-R spectral conversion films, and higher photosynthetic activity under the film was partly attributed to improved electron transport efficiency of PSI and PSII [61, 62]. In lettuce ‘Rouxai’, substituting 60 μmol∙m^–2^∙s^–1^ of G light with R light (in a background of 60 μmol∙m^–2^∙s^–1^ of B light) increased the YPFD by 8% and shoot dry mass by 33% [50]. In this study, as 60 μmol∙m^–2^∙s^–1^ of G light was substituted with R light, the calculated YPFD increased by 6% (Table 1). This relatively small increase in YPFD significantly increased shoot fresh mass (by 26%) and dry mass (by 22%) of lettuce in the presence of FR, but had little effect without FR (Fig 4). Biomass accumulation is closely related to photosynthetic efficiency as well as light capture, which is affected by leaf area and plant architecture [30, 48]. While there were few significant effects of G light on leaf morphology in lettuce regardless of FR presence (Table 2 and Fig 2), substituting G with R in the presence of FR increased leaf number, which was associated with decreases in the FR fraction (or increases in iPPE). Therefore, in the presence of FR, the increase in shoot fresh and dry mass of lettuce by substituting G with R light could be mainly attributed to an increase in leaf number and possibly total leaf area, in addition to a small increase in YPFD. In contrast, substituting G with R light had little to no effect on shoot fresh and dry mass of tomato in this study, regardless of FR light (Fig 4). In tomato, the substitution of G with R light could have decreased light capture efficiency from the decrease in stem elongation, thus offsetting any possible increase in photosynthetic efficiency.

### 4.2. Effects of excluding FR light on plant growth

The promotion effect of FR light on extension growth of stems and leaves, subsequently increasing crop growth and yield, have been reported in lettuce [63, 64], tomato [65, 66], and ornamental seedlings [46]. For example, lettuce ‘Sunmang’ grown at a PPFD of 130 μmol·m^−2^·s^−1^ (provided by 20%B+80%R LEDs) without FR had 33% lower shoot fresh mass and 28% lower shoot dry mass than plants grown with an additional 87 μmol∙m^−2^∙s^−1^ of FR light [63]. In tomato ‘Komeett’, the shoot dry mass when grown at a PPFD of 150 μmol·m^−2^·s^−1^ (provided by 5%B+95%R LEDs) without FR (PPE = 0.88) was 25–29% lower than plants grown with FR (PPE = 0.70, 0.73, 0.80) [65]. In addition, recent studies with FR photons (specifically 700–750 nm) showed they were as effective as light within PAR for eliciting photosynthetic activity [31, 32]. In sunflower, when FR photons were removed from sunlight using an FR filter at a high PPFD (1775 μmol∙m^−2^∙s^−1^) and moderate PPFD (432 μmol∙m^−2^∙s^−1^), the net photosynthetic rate decreased by 6% and 12%, respectively [34]. Not surprisingly, our results show that the exclusion of FR light and the decrease in FR fraction (and increase in iPPE) decreased plant diameter (15–23%) and leaf length (23–33%) of lettuce and plant height (60–71%) and leaf area (10–37%) of tomato (Fig 2). In addition to the suppressed extension growth, when the extended definition of PAR was applied (i.e., 400–750 nm), the spectrum without FR had a 21–26% lower photon flux density (Table 1). Subsequently, without FR, shoot fresh and dry mass decreased by 22–40% and 28–48% in lettuce, respectively, and by 36–57% and 20–44% in tomato, respectively (Fig 4). Together, these results confirm that excluding FR light or decreasing the FR fraction (or increasing PPE) decreases plant biomass independent of PPFD or B light [67, 68]. The data also support the merit of expanding the definition of PAR to 750 nm [31–34].

FR and G light can somewhat similarly induce shade-avoidance responses, including elongation of hypocotyls, petioles, and stems; leaf hyponasty; and decreased leaf pigmentation [41, 42, 44]. However, in arabidopsis, increasing the FR fraction from 0.25 to 0.91 elicited more pronounced petiole elongation than adding 25 μmol·m^−2^·s^−1^ of G light when B light was constant at 25 μmol·m^−2^·s^−1^ [69]. Similarly, in this study with tomato, the magnitude of a decrease in stem elongation and SPAD index of tomato were greater with the exclusion of 60 μmol·m^−2^·s^−1^ of FR than the substitution of 60 μmol·m^−2^·s^−1^ of G with R light, respectively (Fig 3). In addition, the inclusion of FR light increased plant diameter and leaf length and width of lettuce and increased leaf area and shoot fresh and dry mass of tomato, while substituting G with R light had negligible effects. These results indicate that compared to G light, FR light more effectively promotes stem elongation, leaf expansion, and subsequent plant dry mass accumulation. Stronger responses to excluding FR light than substituting G with R light could be at least partly explained by phytochrome-mediated plant responses, which are quantified by the FR fraction and PPE of a light environment [44, 50]. For example, stem length linearly increased as the FR fraction or PPE increased [45, 52]. In this study, the exclusion of 60 μmol m^–2^ s^–1^ of FR decreased the FR fraction by 0.32–0.49 (or increased iPPE by 0.39–0.50), and substituting G with R caused smaller decreases (by 0.01–0.18 or 0.04–0.15, respectively) (Table 1). Therefore, greenhouse films that decrease transmission of FR light will likely have a greater impact on plant morphology, as well as biomass accumulation, than those that convert G photons to R photons.

This study defined FR light (700–800 nm) based on a 100-nm waveband, which is the definition from the American National Standards Institute [21]. We simulated an NIR film applied to a greenhouse with a cutoff near 700 nm by providing plants with different spectra with or without 60 μmol∙m^−2^∙s^−1^ of FR at a peak wavelength of 733 nm. In addition, most of FR photons (94%) in this study were between 700 and 750 nm. The effects of FR light on phytochrome-mediated responses and photosynthesis depend on the wavelengths of FR light. The efficacy of FR photons to inactivate phytochrome is greatest at about 730–740 nm [52]. FR light showed photosynthetic activity within 700–750 nm but not above 752 nm [32, 70]. Thus, most (if not all) of the morphological and plant growth responses observed in this study could be attributed to a narrower waveband of 700–750 nm. Plant responses here support the proposed redefinition of PAR to include FR light at 700–750 nm [31–33].

### 4.3. Other considerations

Different greenhouse covering materials have been developed to block incoming NIR inside greenhouses, including fluid roof covers and NIR-reflecting or NIR-absorbing materials (including plastic films, coatings, and movable screens) [7, 26, 27, 71]. Although these materials selectively block NIR while transmitting PAR, they all reduced PAR transmittance. For example, the PAR transmittances of three types of NIR-reflecting plastic film covers or two types of a fluid-roof cover ranged 62–72% or 59–63%, respectively [72]. The PAR transmittance ranged 73–94% for NIR-reflecting glasses, 85–88% for NIR-filtering polyester films, 77–81% for polyethylene films with NIR-reflecting pigments, 71–75% for polyethylene films with NIR-absorbing pigments, and 76% for a NIR-filtering whitewash [25]. Given that crop yield positively correlates with accumulated photosynthetic light (i.e., the photosynthetic daily light integral) [73], using NIR-blocking greenhouse covering materials could decrease crop growth and yield except when under light-saturating conditions. In some situations though, the benefits of a lower greenhouse temperature (e.g., avoiding heat delay of flowering) could outweigh reduced growth consequences. In our simulation, we assumed a “best case” situation in which exclusion of FR did not decrease PAR.

G-to-R conversion films for greenhouse coverings or luminescent solar concentrators contain luminescent organic dyes, which absorb G (and to a lesser extent, B) photons and fluoresce them as R (and to a small extent, FR) photons. However, the reported values of PAR transmittance and efficacy of G-to-R conversion films vary depending on the material. For example, the range of transmittances of B, G, R, and PAR of G-to-R converting greenhouse films were 66–93%, 57–95%, 97–98%, and 74–95%, respectively, depending on the concentration of fluorescent dye [74]. In a separate study, the range of transmittances of B, G, R, FR, and PAR of G-to-R converting luminescent solar concentrator films were 72–88%, 69–87%, 80–98%, 78–97%, and 74–90%, respectively [12]. Compared to a control film, the spectrum conversion film showed 10% higher transmitted irradiance in R light and 10% lower transmitted irradiance in B and G light [6]. In our study, we assumed a “best case” situation in which there was an equal trade-off between G and R photons, and thus, the PPFD was constant. As more G photons were substituted with R ones, lettuce dry mass increased by 6–29%, but there was no effect on tomato growth. Reduced PAR transmittance and lower efficacy of G-to-R light conversion films can attenuate the benefits of spectral conversion.

A few studies addressed potential decreases in spectral conversion efficacy from photodegradation of luminescent organic dyes. For example, the absorbance of a spectral conversion film decreased by 11% without nano-oxide filters or by 2–8% with nano-oxide filters after one year of exposure to sunlight [12]. In contrast, exposure to the sun for 49 days reduced the conversion efficiency of a film by only 0.2%, indicating that the film was relatively stable [75]. In addition to longevity, a barrier to commercial implementation of spectral conversion films is the cost to purchase, install, and maintain them in a greenhouse setting. Finally, the effects of a modified spectrum on plant quality attributes (e.g., leaf or fruit texture, taste, and postharvest longevity) need to be considered.

### 4.4. Conclusion

We created indoor lighting conditions to simulate “best case” scenarios for greenhouse glazing materials that modify the transmitted solar spectrum: converting G into R photons and almost completely excluding FR light. The simulated conversion of G-to-R photons had relatively small and inconsistent effects on leaf expansion of lettuce and tomato, but it suppressed stem elongation of tomato. In the presence of FR light, substitution of G with R also increased the leaf number in lettuce. Subsequently, the small increases in YPFD and photosynthetic efficiency by substituting G with R light increased lettuce growth in the presence of FR photons, but there was little effect on plant biomass in lettuce without FR photons or in tomato regardless of FR presence. Regardless of modifying the G:R, excluding FR from a light spectrum suppressed leaf expansion and plant growth in both crops. These results suggest that solar spectral conversion from G-to-R light can increase crop productivity in at least some species, including lettuce, while excluding FR photons from sunlight can decrease crop growth and yield. Additional factors, such as decreased PAR transmission, spectral conversion efficiency, photostability, and cost of the greenhouse covering materials should be considered for specific applications.

## Supporting information

S1 File Raw data for Fig 1. The spectra of nine emulated lighting treatments consisting of blue (B, 400–500 nm), green (G, 500–600 nm), red (R, 600–700 nm), and far-red (FR, 700–800 nm) light delivered by light-emitting diodes.
(XLSX)

S2 File Raw data for Table 1. Photon flux density and spectral properties of nine emulated lighting treatments consisting of blue (B, 400–500 nm), green (G, 500–600 nm), red (R, 600–700 nm), and far-red (FR, 700–800 nm) light delivered by light-emitting diodes (LEDs).
(XLSX)

S3 File Raw data for Table 2 & Figs 2–4. (XLSX)

## Abbreviations

B: blue
FR: far red
G: green
LEDs: light-emitting diodes
NIR: near infrared
PAR: photosynthetically active radiation
PPE: phytochrome photoequilibrium
PPFD: photosynthetic photon flux density
R: red
TPFD: total photon flux density
YPFD: yield photon flux density
